# Multi Drug Resistant Tuberculosis in Mosango, a Rural Area in the Democratic Republic of Congo

**DOI:** 10.1371/journal.pone.0094618

**Published:** 2014-04-14

**Authors:** Michel Kayomo Kaswa, Serge Bisuta, Georges Kabuya, Octavie Lunguya, André Ndongosieme, Jean Jacques Muyembe, Armand Van Deun, Marleen Boelaert

**Affiliations:** 1 National Tuberculosis Program, Kinshasa, Democratic Republic of Congo; 2 Institut National de Recherche Bio-Médicale, Kinshasa, Democratic Republic of Congo; 3 Institute of Tropical Medicine, Antwerp, Belgium; Fundació Institut d'Investigació en Ciències de la Salut Germans Trias i Barcelona, Universitat Autònoma de Pujol, CIBERES, Spain

## Abstract

Multidrug Resistant Tuberculosis (MDR-TB) is a serious threat which jeopardizes the worldwide efforts to control TB. The Democratic Republic of Congo (DRC) is one of 27 countries with a high burden of MDR-TB. Data on the magnitude, trends, and the distribution of MDR-TB in DRC are scanty. Kinshasa, the capital city of DRC which accounts for 20% of all TB cases nationwide, is notifying more than 80% of all MDR suspects. We report here a cluster of MDR-TB cases that was investigated in the Mosango health district, in the Bandundu south Province, DRC in 2008. Phenotypic Drug Sensitivity Testing and DNA sequencing were performed on 18 sputum specimens collected from 4 MDR-TB suspects and 5 household contacts. Sequencing data confirmed that the 4 suspects were indeed Rifampicin resistant cases. Sequencing of the *rpoB* gene showed that 3 cases (patients A, B and D) had a single mutation encoding a substitution to 526Tyr, 531Trp and 526Leu respectively. Patient C had a double mutation encoding a change to 531Leu and 633Leu. Two of the investigated cases died within 4 months of a second-line treatment course. Results highlight the need to enhance adequate laboratory services within the country for both clinical as well as surveillance purposes.

## Introduction

Multidrug Resistant Tuberculosis (MDR-TB) defined as the resistance of clinical isolates of *Mycobacterium tuberculosis* strains against Rifampicin (RMP) and Isoniazid (INH) is considered as a serious threat which jeopardizes the worldwide efforts to control Tuberculosis [1]. Conventional methods for diagnosing MDR-TB are slow and cumbersome requiring at least 2 months for test execution and the treatment is complex.

In Low-Income Countries such as the Democratic Republic of Congo (DRC), the challenge posed by MDR-TB is huge. During the two last decades, the history of the DRC has been rife with civil unrest, which has led to the collapse of the health system and the recrudescence of Tuberculosis (TB) and other infectious diseases. DRC, with an estimated population of 66 million, is ranked 11^th^ among the 22 TB High Burden Countries and has an estimated incidence of TB of 327 per 100 000 inhabitants per year [2], [3]. DRC is classified also among the 27 countries with a high burden of MDR-TB [4], but the actual data on the magnitude, trends, and the distribution of MDR-TB in DRC are scanty. In 2008 the World Health Organization (WHO) estimated that the total number of MDR-TB cases in DRC was 5 600 (95%CI: 530–11 000) [4]. However, less than 2% of this estimated number were detected and put on specific treatment during that same year. Kinshasa, the capital city of DRC which accounts for 20% of all TB cases nationwide, is notifying more than 80% of all MDR suspects, and not all of these are laboratory-confirmed (DRC National TB Program data; unpublished). The burden of MDR-TB in the rest of the country is even less well defined as a result of the scarce laboratory infrastructure and some logistical limitations. Extremely long turn-around times for laboratory results to reach the treating clinician increase the risk of the spread of resistant strains.

Because of the need to closely monitor the spread of this problem across DRC and the scarcity of data beyond the capital Kinshasa, we report here a cluster of MDR-TB cases that occurred in the Mosango health district, in the Bandundu south Province, DRC in 2008.

## Methods

### Ethics statement

This study used specimens and data collected in the course of routine patient care and resistance surveillance, performed without ethics review or informed consent. The study was approved by the health authorities of DR Congo. To ensure confidentiality, the data were completely delinked from any personal identifiers before data analysis.

### Settings

During September 2008, the National TB Program (NTP) of DRC was notified by the Chief Medical Officer of Mosango health district about the admission of 2 laboratory-confirmed cases of MDR-TB in the inpatient wards of the Mosango Hospital. Mosango hospital is the third largest tertiary hospital in DRC with a capacity of 541 beds of which 121 are fully dedicated to TB patients. It is located at 420 Km from Kinshasa. In response to this alert, the NTP launched an investigation and sent a team of 1 microbiologist and public health officer (MKK), 1 MDR program officer (SB) and 1 laboratory technician (GK) to Mosango to review the 2 case histories and actively search for other MDR suspects amongst TB cases and contacts. The team reached the hospital in October 2008 and reviewed all available NTP records as well as the hospital admission register and patient files. Patients in retreatment for tuberculosis with Acid-Fast Bacilli (AFB) smear positive at month 3 or 5 were considered as MDR-TB suspects. A contact was defined as a first degree relative of a MDR-TB suspect living in the same household for at least 3 months. All retrieved retreatment cases positive for AFB from Mosango Hospital area were listed, their demographic data, clinical TB history and household contacts information were collected and a list of their close contacts was then established. Individual demographic, clinical and laboratory data were captured in an Excel sheet (version 5.0) and later analyzed by the software Epi-Info 3.5.4 (Centers for Disease Control and Prevention, Atlanta, GA).

### Laboratory procedures

From each suspect and those contacts identified and included in this investigation, 2 sputum specimens were collected to ensure an adequate recovery of *Mycobacterium tuberculosis* on solid medium Löwenstein Jensen (LJ) and followed by detection of RMP and INH resistance by conventional Drug Susceptibility Testing (DST) on solid medium and further DNA sequencing. Firstly, sputum specimens were collected in a Falcon tube containing 5 ml of cetyl-pyridinium chloride (CPC) at 1% and were transported to the National Reference Laboratory (NRL) in Kinshasa for culture and DST on LJ medium. For primary culture, sputum samples were processed according to standard methods previously described [5]: decontamination and processing with NaOH according to a modified Petroff technique [6], with a contact time shortened to 10 min. First Line Drugs (FLD) DST was performed on Löwenstein-Jensen according to the indirect proportion method as described by Canetti et al. [7] with final readings at 6 weeks. Strains appearing to belong to the *Mycobacterium tuberculosis* complex (because of their macroscopic appearance, acid fast staining and slow growth) were tested at the critical concentrations of INH 0.2 µg/ml and RMP 40 µg/ml, besides ρ-nitrobenzoic acid (PNB) for differentiation from non-TB mycobacteria according to international guidelines [8]. Secondly, for each sputum specimen collected, an aliquot was preserved in 50% alcohol to permit additional genetic testing in the Supra National Reference Laboratory (SRL) in Antwerp/Belgium. RMP mutations were defined later and independently of DST result by sequencing of the *rpoB* gene. DNA extracts from clinical specimens were prepared using the automated Boom extraction method as described elsewhere [9]. Detection of *rpoB* mutations, targeting a 1,674-bp region from codon 176 to 672, from which RMP resistance-conferring mutations have been reported, was performed as described previously. A nested PCR with primers *rpoB*geneSAnew (5′-GCAAAACAGCCGCTAGTCCTAGTCCGA-3′) and *rpoB*geneRA (5′-GCGCCATCTCGCCGTCGTCAGTACAG-3′) for the first run, and *rpoB*geneSA and *rpoB*geneRB as inner primers, was used for amplification [10]. All Rifampicin resistance-determining region (RRDR) mutations, plus others previously reported for the *rpoB* gene, were considered potentially relevant for Rifampicin resistance [11], [12]. Mutations were identified by *rpoB* codon number (Escherichia coli numbering) and amino acid substitution [13]. INH resistance was not determined genetically.

## Results

The two index cases mentioned above could not be included in this investigation: one had died before the arrival of the team, and no clinical specimens were available for the other case. The Mosango district TB register listed a total of 24 cases as being in retreatment in October 2008, including the two index cases. We could retrieve 16 (73%) of the 22 suspects within a perimeter of 50 Km around the hospital (the others left an incorrect address, had left the area or lived beyond 50 Km from the hospital). Ten (42%) had clinical symptoms and we collected their sputum for AFB testing. Four of them were AFB positive and were considered as “MDR suspects”. Three of them were on their third TB treatment course, and one patient was taking the second course of first-line drugs. None of them had ever interrupted treatment. The team listed 28 household contacts for these 4 patients. Twenty (71%) contacts were retrieved and screened for TB symptoms. Five (25%) had at least one symptom suggesting TB. Culture, phenotypic DST and DNA sequencing were performed on 18 sputum specimens collected from 4 MDR-TB suspects and 5 household contacts. Table 1 summarizes demographic, clinical and laboratory results of the 9 suspects included in the final analysis. Median age was 28 years (IQR 24–38), 5 (56%) were female. All patient samples were positive in culture (4/4) but none of the samples of their household contacts was positive. Conventional DST showed RMP and INH resistance (MDR) in 2 out of the 4 positive patients and RMP resistance in one. However, sequencing data confirmed that the 4 suspects were indeed RMP-resistant cases. Sequencing of the *rpoB* gene showed that 3 MDR cases (patients A, B and D) had a single mutation encoding a substitution to 526Tyr, 531Trp and 526Leu respectively. Patient C had double mutations encoding a change to 531Leu and 633Leu. Patients B and D died within 4 months of second line drugs (SLD) treatment with Kanamycin, Ofloxacin, Prothionamide, Cycloserine, Pyrazinamide and Ethambutol. Their HIV status was not known. Patients A and C had favorable treatment outcomes under SLD. Recently we reviewed again the TB registers of Mosango health district to assess clinical outcomes of the 36 retreatment cases enrolled in 2008 (not including the 6 MDR patients described above). Treatment outcomes were available for thirty three of the 36: 25 were cured, 4 died, 3 failed and 1 was transferred out.

**Table 1:** Demographic, clinical and laboratory results of MDR suspects (n = 9).

N°	Status	Age	Sexe	Clinical History	N° of FLD course	Culture and DST on LJ in NRL Kinshasa					*RpoB* Sequencing at SRL in Antwerp	Treatment outcomes of SLD assessed in 2011
						SM	Culture	ID	RMP	INH		
A	Case	20	F	F2	3	+	+	MTBC	R	C	His526Tyr(TAC)	Treatment completed
B	Case	29	M	F1	2	++	+	MTBC	R	R	Ser531Trp(TGG)	Died
C	Case	31	F	R2	3	++	+	MTBC	R	R	Ser531Leu(TTG), Arg633Leu (CTC)	Cured
D	Case	38	M	F2	3	++	+	MTBC	S	R	His526Leu(CTC)	Died
A1	Contact	6	F	NA	NA	0	NG	NT				
A2	Contact	22	M	NA	NA	0	NG	NT				
B1	Contact	59	F	NA	NA	0	NG	NT				
C1	Contact	24	F	NA	NA	0	NG	NT				
D1	Contact	41	M	NA	NA	0	NG	NT				

**A1**: First contact of case A; **A2**: Second contact of case A; **B1**: First contact of case B; **C1**: First contact of case C; **D1**: First contact of case D. **M**: male; **F**: female. **F1**: Failure in WHO standard first line treatment regimen for category 1 for new cases; **F2**: Failure in WHO standard first line treatment regimen for category 2 retreatment cases; **R2**: Relapse in WHO standard first line treatment regimen for category 2 retreatment cases. **NA**: Not applicable. **N° of FLD**: Number of first-line drugs. **SM**: smear microscopy results. **NG**: no growth. **ID**: identification. **MTBC**: *Mycobacterium tuberculosis* complex. **NT**: not tested. **R**: Resistant; **S**: sensitive; **C**: contaminated. **SLD**: second-line drugs.

## Discussion

The cluster of MDR-TB cases reported here is to our knowledge the first published account of confirmed MDR-TB from a rural area in DRC. Our findings suggest that MDR-TB is present in Mosango Health district since at least 2008. The DNA sequencing documented molecular mutations of *rpoB* genes conferring resistance in MTBC strains in all 4 suspect patients investigated, but in none of their contacts. Conventional DST performed poorly and missed 2 of these 4 cases of MDR-TB. This is partly explained by contamination of DST but also by highly variable results for RMP sensitivity in the proficiency testing at the Kinshasa NRL. Unfortunately, the delay in diagnosis and appropriate management of the cases led to a high case fatality rate: two of the 4 investigated cases and 1 of the two index cases died. This high case fatality is most likely due to the inadequate retreatment regimen used during several months, as the risk of death with standard short-course chemotherapy is highest when there is resistance to both INH and RMP [14], [15].

The sequencing data did show a set of 4 different drug resistant patterns in the 4 cases suggesting no evidence of a link between these MDR-TB cases. Most likely, the MDR-TB was due to acquired resistance in patients previously treated. Three MDR patients had a strain with a single *rpoB* mutation (526Tyr, 531Trp and 526Leu) and one patient had a strain with a double mutation 531Leu and 633Leu. This double mutation observed in a patient who had been previously treated 3 times with First-Line Drug treatment, is probably due to acquired drug resistance [16]. The mutation 531Leu has been described recently as the most frequent in Kinshasa among relapse and treatment failure TB cases. In a prospective cohort study of TB patients starting retreatment, Van Deun found that this mutation accounts for 63% of all *rpoB* mutations from first recurrence sputum specimens from Kinshasa [17]. Contrastingly, our series consisted of multiple retreatment cases. The MDR-TB patient D had a strain with 526Leu, a mutation that is considered rare by some authors [18]. However the *rpoB* 526Leu, which is part of the group called “disputed” mutations described previously by Van Deun, is rather common in DRC and elsewhere [17], [18]. Of note, conventional DST at Kinshasa NRL failed to detect it [19], [20]. DNA sequencing performed in our study showed the *rpoB* 633Leu in a double mutation, and to the best of our knowledge, this was never described before. Current molecular DST targeting only the RRDR (codons 507–533) is missing it. We doubt its clinical significance, as it has been found only once and accompanying other, more common mutations. If more frequently seen and also in isolation it might be assumed to have significance.

This study has some limitations. The case definition for a suspect case used in the outbreak investigation was rather restrictive and probably allowed to select only a part of all the people at risk of MDR-TB. Secondly, INH resistance was not determined genetically. Even if RMP resistance is widely recognized as a proxy for MDR-TB, the genotypic pattern of INH is important for surveillance and molecular epidemiology of MDR-TB.

Culture of sputum on a solid agar medium could provide more reliable DST results but is extremely lengthy and significantly delays the initiation of adequate therapy [21]. Liquid-growth based methods and Nucleic Acid Amplifications techniques have demonstrated superior performance in many settings [22], [23], [24], [25], [26]. However under field conditions in high TB settings and low income countries, such as DRC, these methods have high technical and logistical requirements that are not easy to meet in a sustainable and accessible way. The latest genotypic techniques might be definitely much easier to set up and run [27], [28]. From a public health perspective, rapid and timely detection of TB cases and strengthened capacity to diagnose cases of drug-resistant TB remain thus global priorities for TB care and control.

## Conclusion

Results observed during this investigation highlight the need to enhance adequate laboratory services in countries confronted with MDR-TB. Fragile states as DRC are facing huge barriers requiring significant investment in laboratory infrastructures and strengthening of human resources. MDR-TB is a serious concern to be considered in all rural settings in Africa and elsewhere, and surveillance of DR-TB should be strengthened globally. A Point-of-Care diagnostic tool, one that can be more readily deployed at the district level would greatly facilitate the timely detection of resistant TB. Its introduction will not only serve diagnosis but also routine surveillance. Sustained SLD provision and adequate case management of MDR-TB patients are moreover essential.
